# Adsorption of the non-steroidal anti-inflammatory drug (ibuprofen) onto biochar and magnetic biochar prepared from chrysanthemum waste of the beverage industry

**DOI:** 10.1039/d3ra01949g

**Published:** 2023-05-15

**Authors:** Yuvarat Ngernyen, Decha Petsri, Kamonchanok Sribanthao, Krittiya Kongpennit, Palita Pinijnam, Rinrada Pedsakul, Andrew J. Hunt

**Affiliations:** a Biomass & Bioenergy Research Laboratory, Department of Chemical Engineering, Faculty of Engineering, Khon Kaen University Khon Kaen 40002 Thailand nyuvarat@kku.ac.th; b Lahan Sai Ratchadaphisek School Lahansai District Buriram 31170 Thailand; c Materials Chemistry Research Center, Department of Chemistry, Center of Excellence for Innovation in Chemistry, Faculty of Science, Khon Kaen University Khon Kaen 40002 Thailand

## Abstract

Biochar and magnetic biochar prepared from chrysanthemum waste of the beverage industry are effective adsorbents for the removal of the non-steroidal anti-inflammatory drug, ibuprofen (IBP), from aqueous systems. The development of magnetic biochar using iron chloride, overcame poor separation characteristics from the liquid phase of the powdered biochar after adsorption. Characterisation of biochars was achieved through Fourier transform infrared spectroscopy (FTIR), thermogravimetric analysis (TGA), N_2_ adsorption/desorption porosimetry, scanning electron microscopy (SEM), electron dispersive X-ray analysis (EDX), X-ray photoelectron spectroscopy (XPS), vibrating sample magnetometer (VSM), moisture and ash content, bulk density, pH and zero-point charge (pH_pzc_). The specific surface area of non-magnetic and magnetic biochars was 220 and 194 m^2^ g^−1^, respectively. Adsorption of ibuprofen was optimised with respect to contact time (5–180 min), solution pH (2–12) and initial drug concentration (5–100 mg L^−1^), with equilibrium being reached in 1 hour, and the maximum ibuprofen removal occurred at pH 2 and 4 for biochar and magnetic biochars, respectively. Investigation of the adsorption kinetics was achieved through application of the pseudo-first order, pseudo-second order, Elovich and intra-particle diffusion models. Adsorption equilibrium was evaluated using Langmuir, Freundlich and Langmuir–Freundlich isotherm models. The adsorption kinetics and isotherms for both biochars are well described by pseudo-second order kinetic and Langmuir–Freundlich isotherm models, respectively, with the maximum adsorption capacity of biochar and magnetic biochar being 167 and 140 mg g^−1^, respectively. Chrysanthemum derived non-magnetic and magnetic biochars exhibited significant potential as sustainable adsorbents toward the removal of emerging pharmaceutical pollutants such as ibuprofen from aqueous solution.

## Abstract 2

Biochar and magnetic biochar prepared from chrysanthemum waste of the beverage industry are effective adsorbents for the removal of the non-steroidal anti-inflammatory drug, ibuprofen (IBP), from aqueous systems.

## Graphical abstract

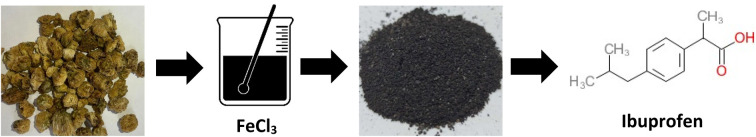

## 1 Introduction

Biochar is a carbonaceous material produced by pyrolysis of biomass in an inert atmosphere or under hydrothermal carbonisation conditions.^1,2^ The type of biomass, production method and conditions all affect the final properties of biochar.^2^ Biochar has received significant attention in applications such as adsorbents and also in soil amendment, both of which aid in mitigating global warming.^3^ Typically, biochar has been observed to have a lower adsorption capacity compared to activated carbon. For example, the maximum removal of phenol from aqueous solution with biochar and activated carbon was 55% and 95%, respectively.^4^ Manfrin *et al.* developed biochar and activated carbon from cigarette waste and applied them to the adsorption of Pb^2+^. The maximum adsorption capacity was 71.42 mg g^−1^ for activated carbon, while biochar was observed to be significantly lower, only enabling a recovery of 23.69 mg g^−1^.^5^ Mahmuda *et al.* also found that activated carbon from pineapple waste had higher adsorption capacity of methylene blue than biochar.^6^ Although activated carbons have proven to be more efficient as adsorbents than biochars, due to their having higher surface area and porosity, the production of activated carbon is complicated, requires additional synthetic processes, and activating agents, thus adding appreciable costs in comparison to biochar production. It has been demonstrated that the application of activating agent adds the greatest cost to the resulting activated carbons.^7^ Therefore, the development of low-cost biochar adsorbents from industrial by-products is still warranted and importantly aids in valorising waste streams.

Non-steroidal anti-inflammatory drugs (NSAIDs) such as ibuprofen (IBP), are an effective over the counter pain reliever and is the world's third most consumable drug.^8^ The release of IBP from industry or through human excretion can pose a serious problem to aquatic life and water treatment.^9^ Therefore, the wastewater needs to be treated for the protection of human health and environmental safety. Numerous studies have investigated IBP removal by using activated carbons from a variety of biomass sources such as biological sludge from beverage wastewater treatment,^10^*Dillenia indica* peels,^11^*Lemna minor*,^12^ coconut shell,^13^ and cork waste.^14^ In addition, multiwall carbon nanotube,^15^ mesoporous carbon CMK-3,^16^ carbide derived carbon (CDC),^17^ zeolite-rich composite,^18^ sediment,^19^ polymeric resin,^20^ or polyacrylonitrile grafted palm seed powder,^21^ were also used as adsorbent for remove IBP from solution. However, biochars and modified biochars can be used as adsorbents for this application. For example, Salem and Yakoot used rice straw-based biochar to study adsorption kinetics and mechanism of IBP removal.^22^ Du *et al.* investigated IBP removal by *Alternanthera philoxeroides*-based biochar and found that biochar with surface area of 858 m^2^ g^−1^ had maximum adsorption capacity of 172 mg g^−1^.^23^ Recently, Patel *et al.* developed walnut shell activated biochar using H_3_PO_4_ immersion and the resulting biochar with surface area of 686 m^2^ g^−1^ showed maximum adsorption capacity of 69.7 mg g^−1^.^24^ Moreover, biochars such as two wood-waste biochars,^25^ coffee bean husk biochar,^25^ biochar derived from mung bean husk,^26^ date seeds,^27^ and biochar from sugarcane bagasse,^28^ have been modified/synthesised with either steam or H_3_PO_4_ to yield activated biochar that also demonstrate some significant promise. This demonstrates that a variety of biomass feedstocks can be used for the production of carbonaceous materials including biochar for the removal of emerging organic pollutants from aqueous waste streams. However, bio-based industrial wastes are low value and can contribute to waste disposal problems. Chrysanthemum is the world's second most economically important floricultural crop, following rose.^29^ It is a popular ingredient in many types of beverages and offers several health benefits, but significant waste residues are produced during the extraction and beverage production process. After boiled with water, all parts of chrysanthemum remain as solid low value waste stream. Utilisation of such wastes to produce low-cost biochar with high adsorption capacities would be advantageous, it would also aid in promoting industrial symbiosis and developing a circular bio-based economy. However, powdered biochar adsorbents are challenging to remove from the solution may hinder the large-scale application of these materials in water treatment, as such any development of new biochar adsorbents should aim to overcome such disadvantages.

An effective strategy to solve this problem is to introduce ferromagnetic elements into the biochar matrix, thereby creating a magnetic biochar with easily for separation. The most common method utilised to prepare magnetic biochars is the “impregnation–pyrolysis” process, where biomass is impregnating with iron ions and is then subjected to pyrolysis, thus yielding a magnetic biochar. Other synthetic methods for magnetic biochar production include co-precipitation, reductive co-deposition, hydrothermal carbonisation, ball-milling (solvent-free mechanical mixing of biochar and iron oxides), direct pyrolysis of biomass/metal salts (also called the molten salt method), cross-linking of biochar and iron oxides and microwave-assisted pyrolysis.^30^ The impregnation–pyrolysis method has become a popular process due to its simplicity, easy of control, and stable combination of magnetic particles and biochar.^31^ Magnetic biochars have been used to remove heavy metals (Cr(vi),^32^ As(v),^33^ Cd(ii),^34^ Pb(ii)^34^), dyes (methylene blue,^35^ methyl orange^36^), nutrient (phosphorus^37^), organic compounds (phenol,^38^ pentachlorophenol^39^) and other contaminants (nitrate,^40^ fluoride^40^), whereas there is a limit research has focussed on their use as adsorbents for IBP removal.^41^

Herein, this work demonstrates the first reported synthesis of biochar and magnetic biochar by impregnation–pyrolysis method from chrysanthemum waste and its application in IBP removal. The biochars are characterised by Fourier transform infrared spectroscopy (FTIR), thermogravimetric analysis (TGA), Brunauer–Emmett–Teller (BET) surface area and porosity, scanning electron microscopy (SEM), electron dispersive X-ray analysis (EDX), X-ray photoelectron spectroscopy (XPS), X-ray diffraction (XRD) and their physico-chemical properties including moisture content, ash content, bulk density, pH and pH at the point of zero charge (pH_pzc_) are also investigated. The effects of contact time, pH and initial concentration on IBP removal were studied, as too were the application of adsorption isotherm and kinetic models.

## 2 Materials and methods

### 2.1 Biochar production

**Fig. 1:**
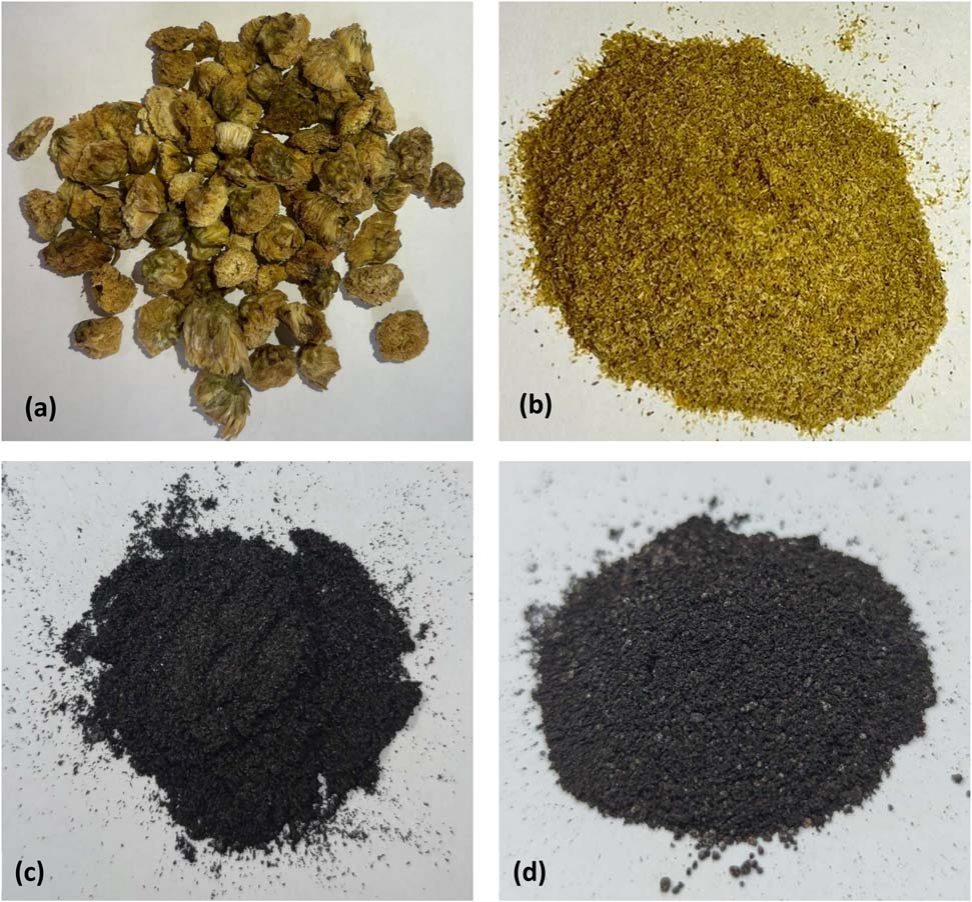
Chrysanthemum wastes and chrysanthemum-based biochars (a) original form, (b) after milled and sieved, (c) non-magnetic and (d) magnetic.

The chrysanthemum wastes (Fig. 1(a)) were obtained from ICHITAN Group Public Company Limited. The material was milled by food processor and sieved with standard sieve of mesh no. 100 (150 μm) and no. 60 (250 μm) to obtained chrysanthemum powder (Fig. 1(b)). 20 g of chrysanthemum powder was pyrolyzed at 800 °C for 90 min under a 200 mL min^−1^ flow of nitrogen. The resulting chrysanthemum biochar was obtained and named CB (Fig. 1(c)).

The magnetic biochar was synthesized as follow: 20 g of chrysanthemum powder was stirred in ferric chloride solution (10 g FeCl_3_·6H_2_O and 150 mL distilled water) for 12 hours. Subsequently, the mixture was dried at 105 °C for 24 hours in an oven. The dried sample was pyrolyzed under the identical conditions to the biochar, the resultant chrysanthemum magnetic biochar was named CMB (Fig. 1(d)). The yield of CB and CMB were calculated based on the final mass of biochar divided by the original mass of raw material.

### 2.2 Biochar characterisation

Fourier transform infrared (FTIR) analysis of raw material and the resulting biochars was achieved using an ALPHA II (Bruker) with spectral range from 500 to 4000 cm^−1^. The spectra of chrysanthemum were analysed using ATR mode, while biochars particles were mixed with KBr to form a KBR disc. FTIR spectra with a resolution of 4 cm^−1^ was collected over an average of 32 scans.

Thermogravimetric analysis (TGA) of samples were performed using Shimadzu DTG-60H with a heating rate of 10 °C min^−1^ under nitrogen atmosphere at a constant flow rate of 50 mL min^−1^ and heating from room temperature to 950 °C.

The pore characteristics were analysed using a gas adsorption apparatus (ASAP 2460, Micromeritics). The Brunauer–Emmett–Teller (BET) equation was used to calculate surface area. The total pore volume was estimated from a single point of N_2_ adsorbed at a relative pressure (*P*/*P*^o^) of about 0.99. The micropore volume was deduced using Dubinin–Radushkevich (DR) methods. The average pore diameter and pore size distribution were determined with the Barrett–Joyner–Halenda (BJH) method applied to the desorption branch of adsorption–desorption isotherm.

The surface morphology of biochars was analysed using scanning electron microscopy (SEM) and electron dispersive X-ray analysis (EDX) (FEI, Helios NanoLab G3 CX). The surface composition, element content and chemical state of the elements in the samples were analysed by X-ray photoelectron spectroscopy (XPS) (PHI 5000 Versa Probe II, Ulvac-PHI Inc). A D8 ADVANCE X-ray diffractometer (Bruker) was also used. For magnetic properties measurement, the samples were investigated using a vibrating sample magnetometer (VSM) (VersaLab) at room temperature.

Moisture content of biochar is determined according to ASTM D2867-70 (Standard Test Methods for Moisture in Activated Carbon) by weighing before and after heating at 150 °C for 3 h in an oven. For determination of ash content, the ASTM D2866-11 (Standard Test Method for Total Ash Content of Activated Carbon) was applied. Typically, 1 g of biochar was placed in a crucible and heated at 650 °C for 1 h in a muffle furnace. Percent ash was calculated by weight of remaining solid divided by weight of original sample, then, multiple by 100. Bulk density was determined follow the procedure described by Yakout *et al.*^42^ The biochar was added to a 10 mL graduated cylinder and tapped constantly until no change in volume was observed. The bulk density was calculated from weight of sample divided by the volume of packed sample. The pH of each biochar sample was measured by refluxing the sample in 100 mL of deionized water. Biochar 1 g was added to refluxing water for 5 h, after which additional 100 mL of deionized water was added and left to cool to room temperature. The pH of the mixed biochar and water slurry was then recorded by pH meter (ST3100-F, OHAUS). The pH_pzc_ was measured by pH drift method. The determination of pH_pzc_ was conducted by adjusting pH of 50 mL 0.01 mol L^−1^ NaCl solution to a value between 2 and 12. The biochar 0.15 g was added and the final pH was measured after 48 h under agitation. The pH_pzc_ of biochar is the point when initial pH equal to final pH.

### 2.3 Drug adsorption

The IBP solution was prepared from distilled water containing 10% vol methanol (99.9%, RCI Labscan) to increase the solubility of IBP. The kinetics were studied at room temperature by adding 0.01 g of biochar in an Erlenmeyer flask containing 50 mL of 30 mg L^−1^ IBP solution for different durations, between 5–180 min. The IBP concentrations were subsequently determined by UV-visible spectrometry (Analytik Jena AG) at 222 nm. The IBP uptake or *q*_*t*_ (mg g^−1^) was calculated from equation:
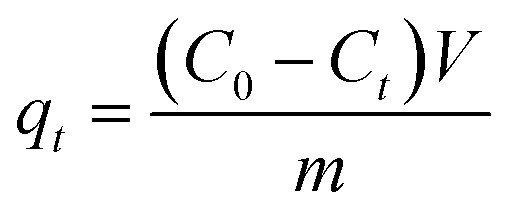
where *C*_o_ is the initial IBP concentration (mg L^−1^), *C*_*t*_ is the IBP concentration at time *t* (mg L^−1^), *V* is the IBP solution volume (L) and *m* is the weight of biochar (g).

The pH variation studies were performed at room temperature and constant equilibrium time obtained from kinetics experiment. The IBP solution (30 mg L^−1^) was prepared in different pH ranges between 2–12, using 0.1 M HCl and NaOH. The isotherms of IBP adsorption on both biochars were studied at room temperature and optimum pH from previous experiment. The amount of biochar of 0.01 g, was introduced in 50 mL IBP solutions of varying concentrations between 5–100 mg L^−1^ using equilibrium time from kinetics experiment.

## 3 Results and discussion

### 3.1 Characterisation of chrysanthemum and biochars

**Fig. 2:**
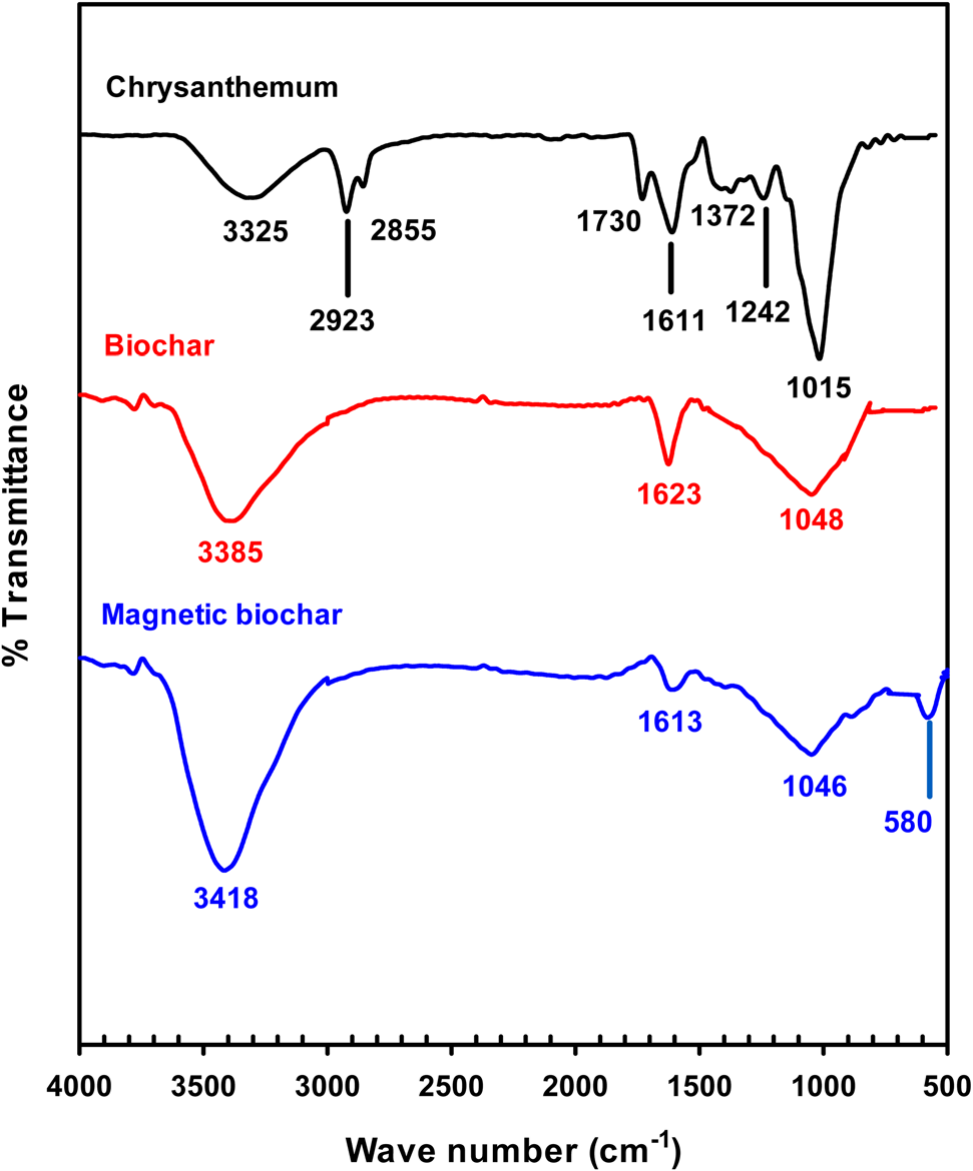
FTIR spectra of chrysanthemum, biochar and magnetic biochar.

FTIR spectrum of raw material, original biochar and magnetic biochar are shown in Fig. 2. The spectrum shows broad band at 3000–3500 cm^−1^ in all samples due to the presence of O–H groups.^43^ For chrysanthemum, bands at 2923 and 2855 cm^−1^ characteristic of aliphatic C–H stretching, which supported by the presence of band at 1372 cm^−1^, assigned to aliphatic C–H folding.^44^ The absorption bands at 1730 and 1242 cm^−1^ corresponding to the stretching of carbonyl C=O and the stretching deformation of C–O and phenolic O–H groups, respectively.^44^ These bands are observed to decrease in intensity or even disappear following pyrolysis and formation of the biochar and magnetic biochar. This is due to extensive dehydration and decarboxylation reactions that take place during the degradation of the original materials by the pyrolysis process. The bands at 1611, 1623 and 1613 cm^−1^ for all samples reflected the presence of C=O functional group on surfaces.^44^ The peaks at 1015 cm^−1^ of chrysanthemum shifted to 1048 and 1046 cm^−1^ for biochar and magnetic biochar represents C–OH bond stretching.^45^ The peak at 580 cm^−1^ is assigned to Fe–O of iron oxides.^46^ Therefore, confirming the production of iron oxides onto the magnetic biochar.

**Fig. 3:**
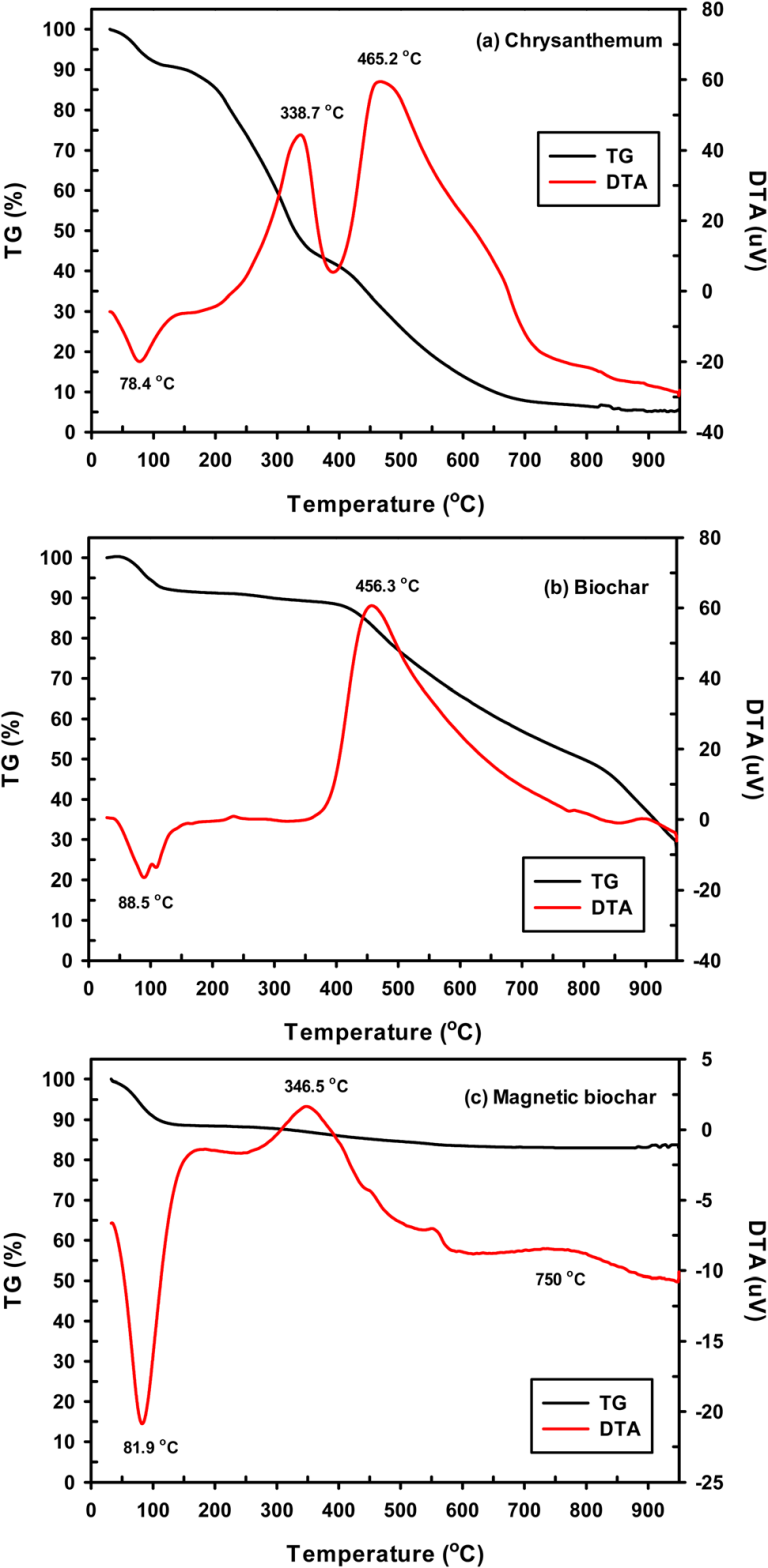
TG/DTA curves for (a) chrysanthemum, (b) biochar and (c) magnetic biochar.

Fig. 3 shows the thermogravimetry (TG) and differential thermal analysis (DTA) curves for the chrysanthemum and two biochars. Three significant peaks can be observed in the DTA curve of chrysanthemum (Fig. 3(a)): a first endothermic peak at 78.4 °C corresponding to the removal of water and light volatile components at temperature lower than 120 °C,^47^ a second exothermic peak at 338.7 °C referred to degradation of hemicellulose,^47^ and a third exothermic peak at 465.2 °C ascribed to lignin and cellulose decomposition.^47^ The second peak was not observed for the biochar (Fig. 3(b)), indicated that the hemicellulose had been degraded during pyrolysis process. For magnetic biochar (Fig. 3(c)), the mass losses at around 750 °C could be explained by the reduction of magnetite Fe^3+^ to Fe^2+^ by the biochar following the reactions (1) and (2).^47^ This agrees with the observation of Fe–O bond from FTIR technique.

1 C_*n*_H_*n*_O → C + volatiles (CO_*x*_, H_2_O, organics)

2 Fe_3_O_4_ + *n*C → 3FeO + *n*CO_*x*_

According to TG results, mass loss in the region up to 100 °C was due to the removal of adsorbed free water, corresponding to about 10% of the total mass in all samples. The mass loss from the TGA of chrysanthemum plateaued at 700 °C, therefore it was anticipated that pyrolysis at 800 °C would result in complete biochar formation. TGA results also demonstrated that the magnetic biochar exhibited better thermal stability than the original biochar. This is due to the activation effect during pyrolysis observed by adding the FeCl_3_ to the biomass.

**Fig. 4:**
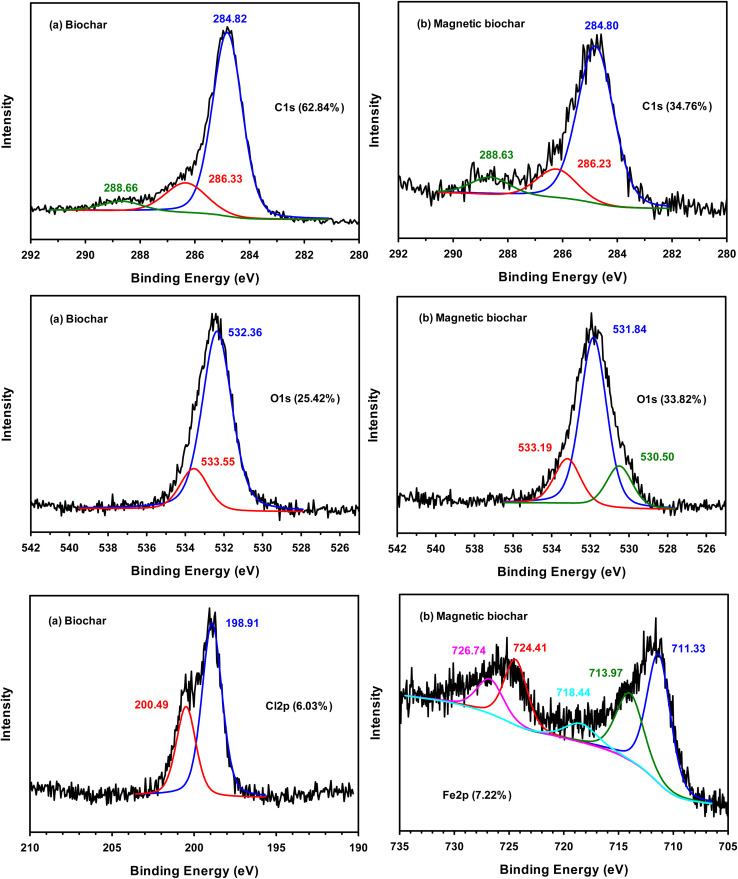
XPS spectra of (a) biochar and (b) magnetic biochar.

The XPS spectra of biochar determined the surface elemental composition to be comprised of C 1s (62.84), O 1s (25.42%), Cl 2p (6.03%), S 2p (3.69%) and Ca 2p (2.02%). S and Ca present in biochar likely comes from the raw biomass material. This is supported by EDX analysis of raw material which revealed that chrysanthemum contained mainly C (70.30%) and O (26.42%) and small amount of S (0.09%) and Ca (0.28%). For magnetic biochar, spectra included C 1s (34.76), O 1s (33.82%), Cl 2p (12.75%), Fe 2p (7.22%), K 2p (4.51%), P 2p (3.09%), Si 2p (2.13%) and Ca 2p (1.72%). These results clearly indicated that after treatment with FeCl_3_, Fe appear in magnetic biochar char, while Cl increased from 6.03% of biochar to 12.75% of magnetic biochar. Fig. 4 shows the individual XPS spectra of C, O, Cl and Fe obtained from both biochars. The main peak at 284.8 eV in the carbon spectra of both biochars is assigned to sp^3^ carbon of C–C, while more limited sp^2^ carbon of C=C is observed at 284.5 eV. Signals in the XPS at 286.3 and 288.6 eV are related to C=O and O–C=O, respectively.^44^ The O 1s spectra of biochar can be deconvoluted to two components: –C=O group at 532.3 eV and C–OH or C–O–C groups at 533.5 eV.^48^ In O 1s spectrum of magnetic biochar, at 531.8 eV represented the Fe–O–C bond, which suggested a strong interaction between Fe_3_O_4_ and biochar.^49^ The peak at 530.5 eV is lattice oxygen in metal oxides (O^2−^).^50^ The peak at 533.2 eV was interpreted as C–O.^49^ The XPS spectrum of Cl 2p of biochar can be deconvoluted into two peaks at binding energies of 198.9 and 200.4 eV. The signal at 200.4 eV was attributed to Cl–C bonds,^51^ while the 198.9 eV peak is assigned to alkali chloride.^52^ For the Fe 2p of magnetic biochar, the peak at 711.3 eV represents Fe^3+^ in Fe_3_O_4_.^49^ Cao *et al.* reported that a peak at 718.4 eV was ascribed to a Fe 2p_3/2_ satellite peak and peak at 724.4 eV attributed to Fe 2p_1/2_ of γ-Fe_2_O_3_.^53^ Finally, the peaks with a binding energy of 713.9 and 726.7 eV corresponded to Fe^4+^.^54^

**Fig. 5:**
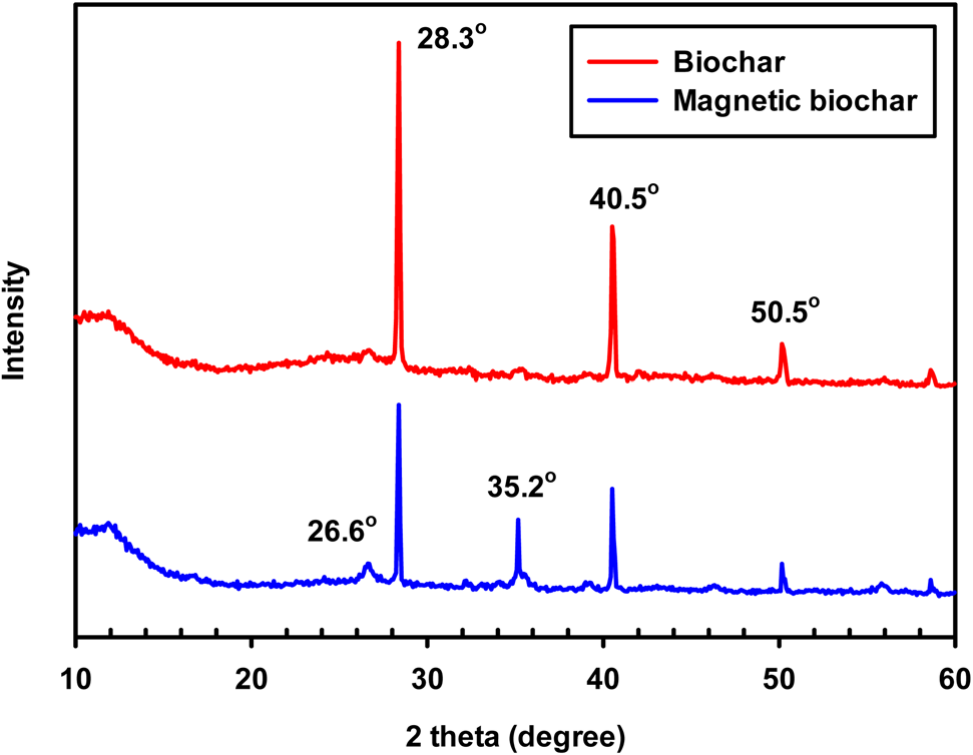
XRD patterns of biochar and magnetic biochar.

XRD analysis was used to evaluate the degree to which the samples were crystalline or amorphous. When a substance is crystalline, well-defined peaks can be observed, whereas non-crystalline or amorphous materials exhibit broad peaks.^55^ XRD patterns of the chrysanthemum biochar and the magnetic biochar (Fig. 5) both indicate the presence of crystalline inorganic minerals such as SiO_2_ (quartz, 2*θ* = 26.6°), KCl (sylvite, 2*θ* = 28.3° and 40.5°) and CaCO_3_ (calcite, 2*θ* = 50.5°).^55^ The presence of these inorganic minerals in the magnetic biochar could also aid to promote the IBP adsorption. Hillerström *et al.*^56^ and Wang *et al.*^57^ demonstrated that it was possible to utilise mesoporous SiO_2_, and CaCO_3_ microparticle to adsorbed IBP, respectively. The XRD peak of magnetic biochar at 35.2° indicate the presence of magnetic FeO·Fe_2_O_3_ crystallite.^58^ For the biochar, a broad signal is observed between a 2*θ* of 20–30° corresponding to amorphous carbon. However, a more pronounced signal at a 2*θ* of 26.6° can be observed in the magnetic biochar corresponding to the development of a graphitic structure and C=C in the biochar. This demonstrates that the addition of ferric chloride (FeCl_3_) during the pyrolysis process leads to the formation of a magnetic biochar.

**Fig. 6:**
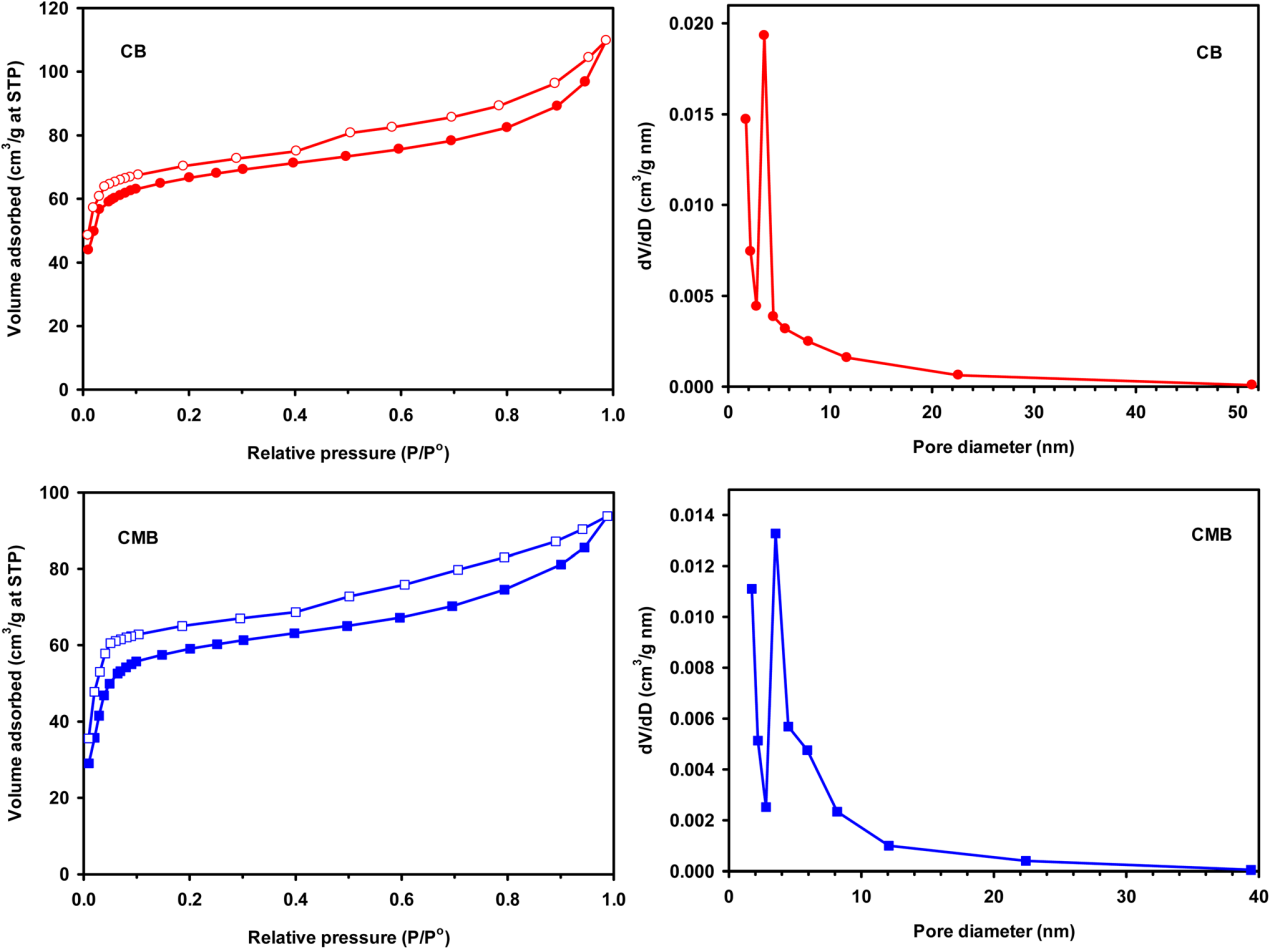
N_2_ adsorption–desorption isotherms and BJH pore size distribution curves of biochar (CB) and magnetic biochar (CMB).

Surface area and pore size distribution of the material plays a significant role in adsorption. BET surface area analysis is based on the monolayer adsorption of nitrogen gas on the surface of the adsorbent, while the pore size is calculated based on pore filling pressures.^59^Fig. 6 shows the nitrogen adsorption–desorption isotherms and the pore size distribution curves of the biochar and magnetic biochar.

Both cases illustrate Type IV adsorption isotherms, showing a hysteresis loop, clearly demonstrating the presence of mesoporosity. The hysteresis loops of biochars were Type H4, which showed the existence of micro–mesoporous composite structure. The desorption branch step down as compared to adsorption branch due to cavitation induced evaporation (the liquid trapped in the pores desorbs through the spontaneous nucleation of a gas bubble). The lower limit of hysteresis loop depends on shape of the micropores or mesopores. Such adsorption–desorption curve indicates that micropores are initially filled, then multilayer type of physisorption and capillary condensation takes place.^59,60^ Using the BJH method and the desorption branch of the nitrogen isotherm, the pore size distribution was calculated, which was predominately less than 10 nm in size, with an average pore diameter for CB and CMB of 3.09 and 2.99, respectively (Fig. 6 and Table 1). This also supports the presence of mesoporosity.

**Table 1:** Comparison of the pore properties of chrysanthemum biochar

Adsorbent	*S*_BET_ (m^2^ g^−1^)	*V*_micro_ (cm^3^ g^−1^)	*V*_meso_ (cm^3^ g^−1^)	*V*_total_ (cm^3^ g^−1^)	*D*_avg_ (nm)
CB	220	0.116 (68%)	0.054 (32%)	0.170	3.09
CMB	194	0.114 (79%)	0.031 (21%)	0.145	2.99

BET surface area of CB (220 m^2^ g^−1^) was higher compared to CMB (194 m^2^ g^−1^). This indicated that the introduction FeCl_3_ on the biochar surface did not enable the formation of greater porosity and additional surface area for adsorption, but in fact the Fe particles may have blocked the pores of the biochar. This was supported by the low pore volumes observed for CMB as compared to that of CB, which changed from 0.170 to 0.145 cm^3^ g^−1^. Xu *et al.* suggested that the Fe particles on the surface of biochar were overlapped and piled up with each other, resulting in the reduction of the pore volume and decreasing the specific surface area.^43^ The decrease in pore diameter also supported the theory that Fe particles were present within the porous structure.

In addition, chrysanthemum biochar prepared from this work had higher or comparable surface area that other biochars reported in literature, for example medium density fibreboard powder residue (2.3 m^2^ g^−1^),^37^ banana (4.7 m^2^ g^−1^),^61^ cassava (13.2 m^2^ g^−1^),^61^ swine manure (3.4–63 m^2^ g^−1^),^62^ rice straw (63–76 m^2^ g^−1^),^22^ industrial kraft lignin (111 m^2^ g^−1^),^63^ and spent mushroom (16–290 m^2^ g^−1^).^64^ However, differences in textual properties of resulting biochars may also be due to the preparation conditions, such as pyrolysis temperature, pyrolysis time or the additional steps of using activation gas like steam. Moreover, the European Biochar Certificate standard (EBC) states that the biochar should have the surface area larger than 150 m^2^ g^−1^.^65^ Therefore, chrysanthemum waste from the beverage industrial is a suitable feedstock for biochar production.

**Fig. 7:**
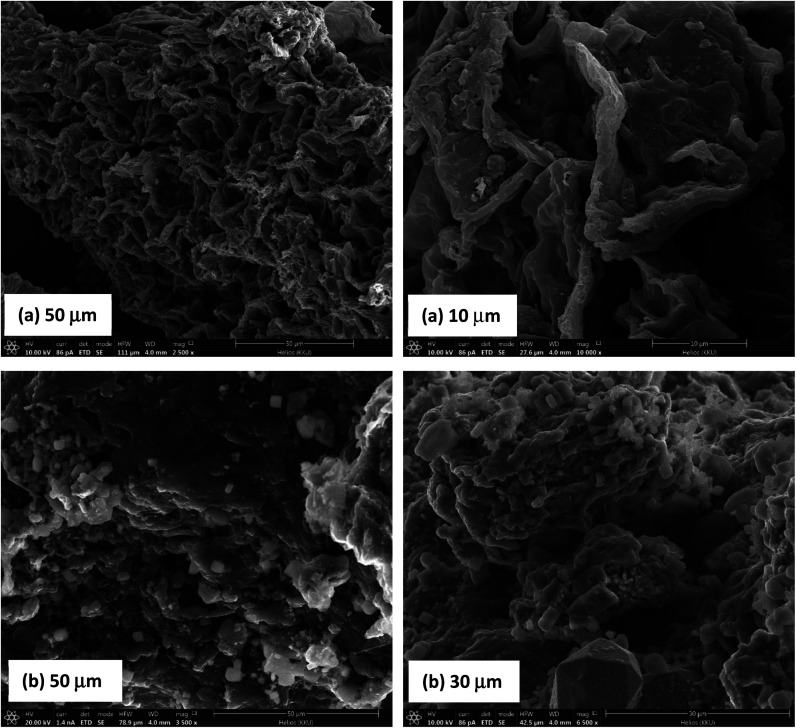
SEM images of (a) biochar and (b) magnetic biochar.

**Fig. 8:**
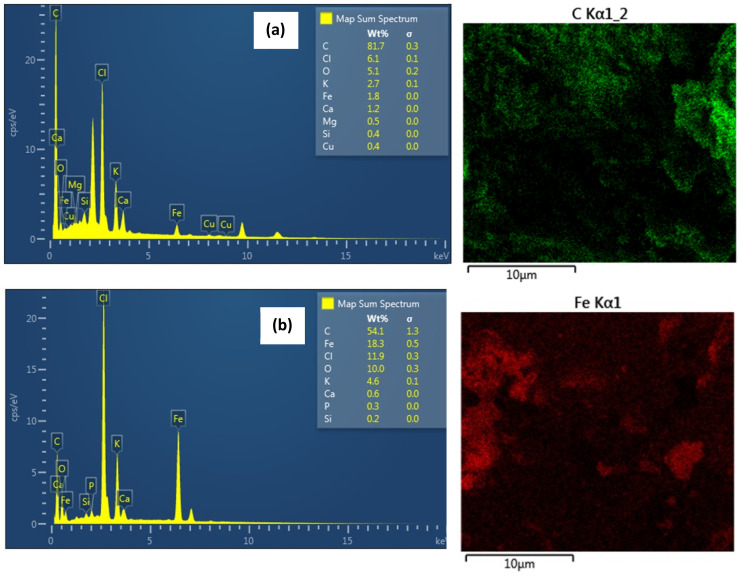
EDX analysis and distribution of C and Fe in (a) biochar and (b) magnetic biochar.

The surface morphology of biochar and magnetic biochar as observed by SEM can be seen in Fig. 7. The surface of both samples demonstrates a significant number of cavities, indicating highly porous structure with large surface area and pore volume. EDX results indicate that the surface of biochar is predominately composed of C (81.7 wt%) (Fig. 8(a)). After introducing ferric chloride (FeCl_3_) into the biochar, C decreased to 54.1 wt% and Fe appear about 18.3 wt% (Fig. 8(b)). The amount of Cl present in the magnetic biochar (11.9 wt%) is significantly higher than non-magnetic biochar (6.1 wt%) due to the ferric chloride. These results are in good agreement with XPS observations. Some inorganic elements such as K, Ca, Mg, Si and P were present in both biochars and likely to originate from the raw material.

**Fig. 9:**
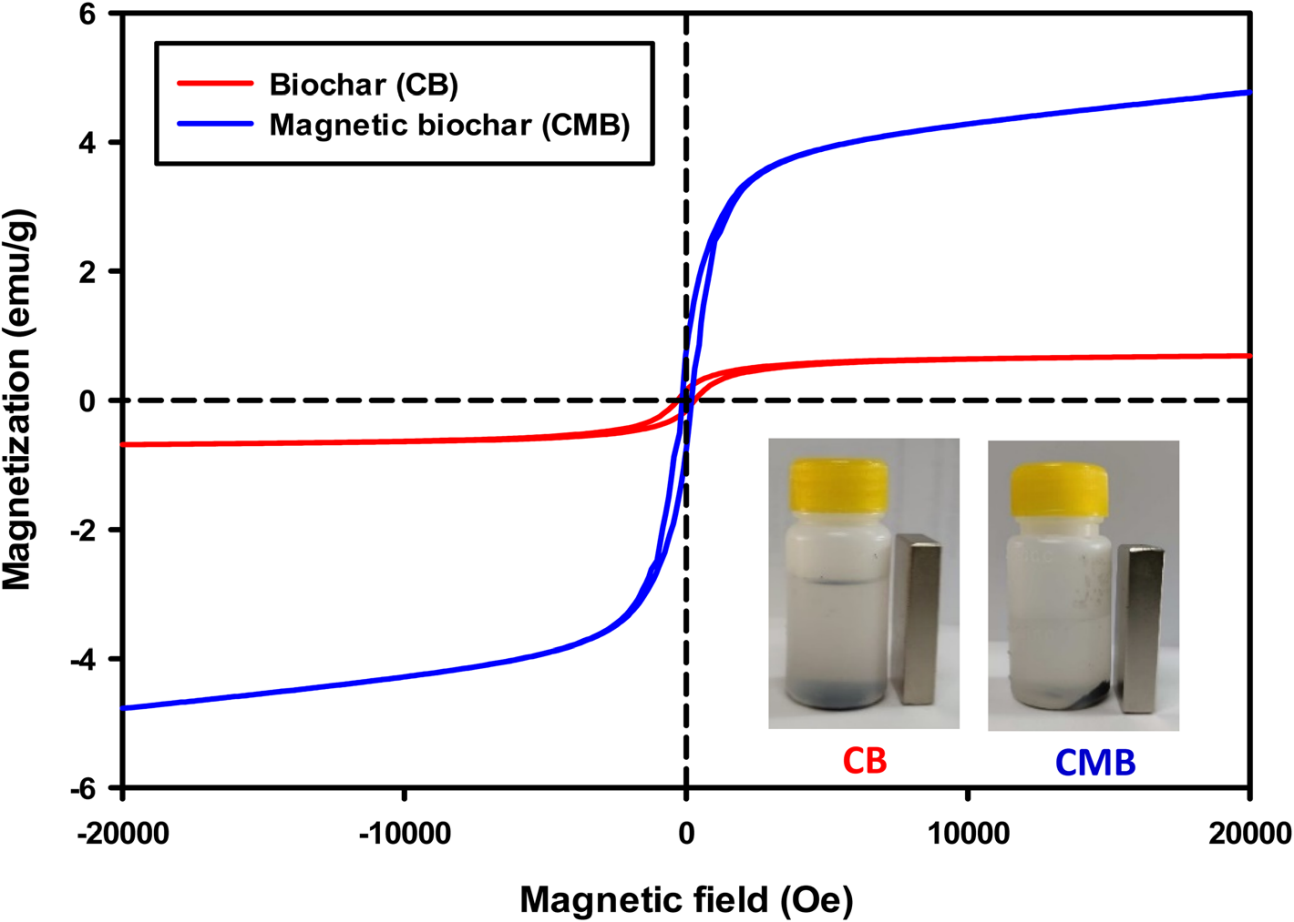
Magnetic hysteresis curve of biochar and magnetic biochar and image of IBP solutions after attracting by magnet.

The magnetic properties of both biochars at room temperature is shown in Fig. 9. From the curve, the magnetization of the chrysanthemum magnetic biochar is higher compared to the pure one which due to the introduce of ferric chloride. The small value of magnetization for original biochar also in agree with EDX result (Fe 1.8 wt%). To test the magnetic separability of biochars from solution, a magnet was put near the container. As displayed in Fig. 9, CMB stick on the surface of container near the magnet, whereas no any happen with CB although it had small magnetization. This demonstrates the magnetic sensitivity of CMB than CB, displaying a potential advantage for the separation, recovery and reuse of adsorbent as well as catalyst.

The yield of biochar and magnetic biochar were 32.5 and 40.5 wt%, respectively (Table 2). Similar results were reported for other biomass-based biochar such as palm oil mill residues (26.3–32.1 wt%),^66^ human manure (30.6–51.9 wt%),^67^ rice husk (35–55 wt%),^68^ and water hyacinth (35.9–62.2 wt%).^69^ A weight loss between 60–68% were observed at high pyrolysis temperature (800 °C) was attributed to a greater release of volatile matter. Moreover, the magnetic biochar had higher yields than non-magnetic biochar. This observation is consistent with the work of Tu *et al.*^70^ The moisture content of the biochars decreased from 10.68 for the original biomass to 9.12 and 9.15 wt% for biochar and magnetic biochar, respectively. This is in agreement with the work of Tsai *et al.*,^62^ Liu *et al.*,^67^ Shariff *et al.*^71^ and Łapczyńska-Kordon *et al.*^72^ The ash content of the biochar increased from 12.50 to 18.26 wt% when compared with the feedstock, this is due to the mineral content of ash, which remains in biochar after the pyrolysis process, while organic matter is lost. Moreover, the surface loaded iron oxide significantly increased the ash content.

**Table 2:** Physico-chemical characteristics of chrysanthemum and biochars

Properties	Chrysanthemum	Biochar	Magnetic biochar
Yield (wt%)	—	32.5	40.5
Moisture content (wt%)	10.68	9.12	9.15
Ash content (wt%)	12.50	18.26	29.39
Bulk density (g cm^−3^)	0.25	0.23	0.26
pH	—	3.08	4.09
pH_pzc_	—	2.40	4.40

The experimental results of the bulk density of chrysanthemum and biochars indicate that the biochars had lower bulk density than raw material (Table 2). This may be related to the pyrolysis process causes a release of volatiles and subsequently causes development of porosity. Moreover, the magnetic biochar shows higher bulk density than that of original biochar, due to the presence of iron in the structure. However, the bulk density of chrysanthemum biochars are lower when compared to other biochars reported in the literature, for example water hyacinth (0.29–0.35 g cm^−3^),^69^ cauliflower leaves (0.37 g cm^−3^),^73^ orange peel (0.46 g cm^−3^),^73^ or pea pod (0.65 g cm^−3^).^73^

**Fig. 10:**
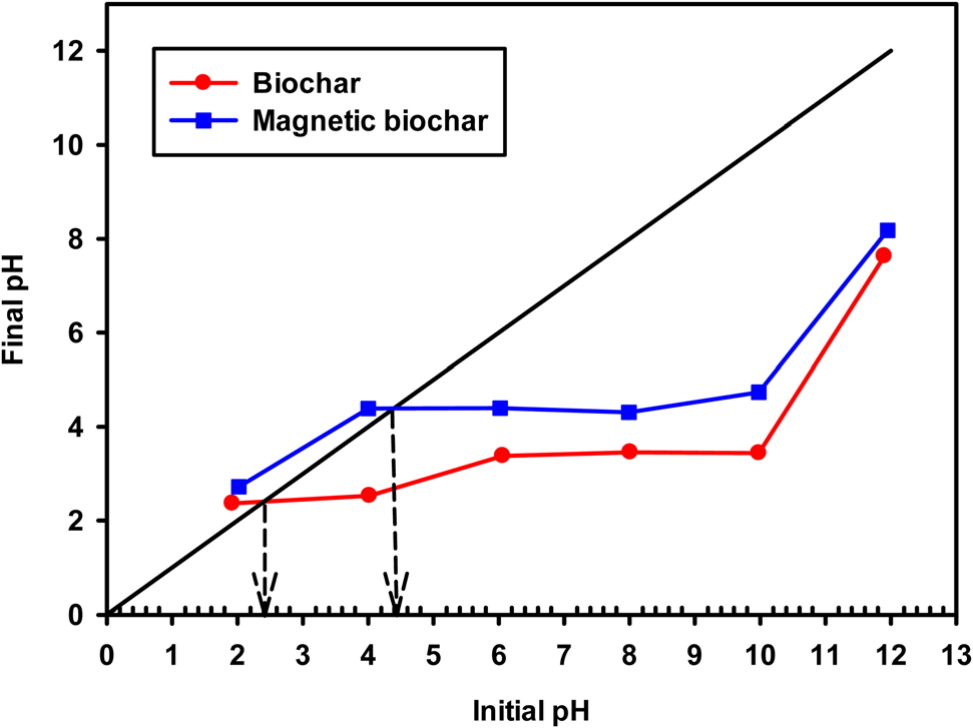
Plot of pH_pzc_ for biochar and magnetic biochar.

The pH and pH_pzc_ of the adsorbent directly impact the adsorption process and the biochars obtained in this study had acidic character (pH 3.08 and 4.09 for non-magnetic and magnetic biochar, respectively). This may be due to the presence of carboxylic acid and hydroxy functional groups.^74^ The pH_pzc_ is defined as the pH in which the surface charge density of the adsorbent is zero. Therefore, when the pH of the solution containing the adsorbate is lower than pH_pzc_, the adsorbent surface has a predominantly positively charged and as such it is favourable to adsorb negatively charged species. In solutions with pH values above pH_pzc_, the net surface charge is negative and it is likely to adsorb positively charged species. pH_pzc_ was 2.40 and 4.40 (Fig. 10 and Table 2) for biochar and magnetic biochar, respectively.

### 3.2 Adsorption experiments

#### 3.2.1 Effect of contact time and kinetic analysis

**Fig. 11:**
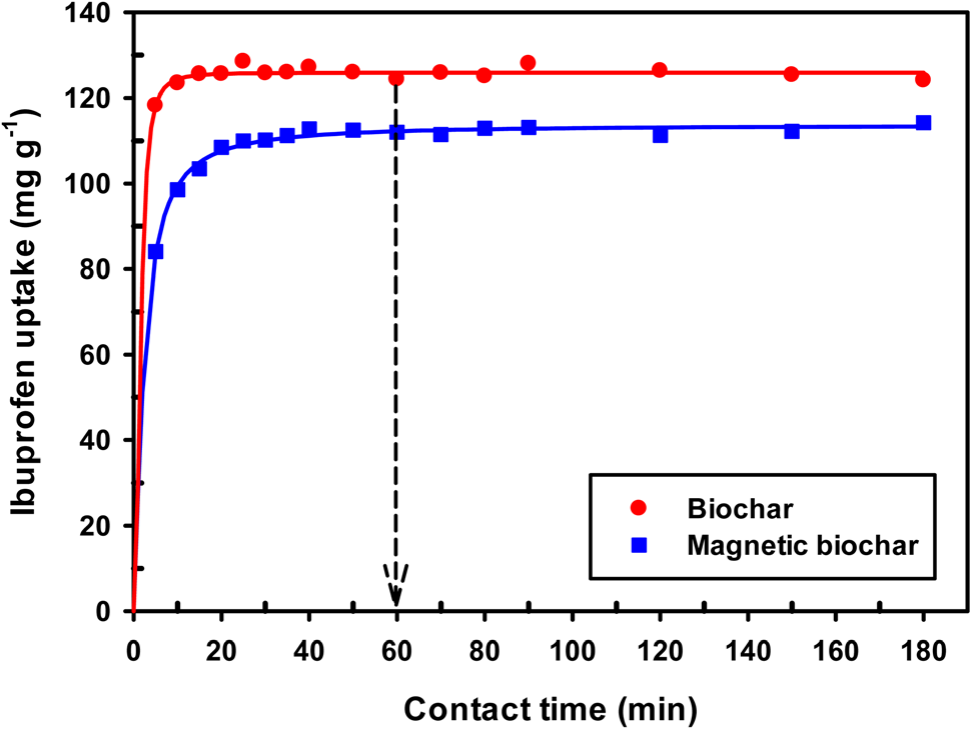
Effect of contact time on adsorption of IBP (room temperature; *C*_o_ = 30 mg L^−1^; dosage 0.01 g/50 mL).

The influence of contact time on the adsorption of IBP can be observed in Fig. 11. Rapid adsorption is observed during the initial 30 minutes, after which adsorption plateaus and reaches equilibrium. The rapid initial adsorption is due to the highly accessible active sites, whereas the observed plateau corresponds to equilibrium with IBP molecules entering and leaving the active sites at the same rate. However, magnetic biochar had slightly lower adsorption capacity than original biochar. This may be due to lower surface area and iron oxides obstructing the IBP molecules from adsorbing on the adsorption sites of the biochar. In conclusion, it is appropriate to choose 60 min as the equilibrium time for subsequent experiments.

The experimental data was further analysed with various kinetic models including the pseudo-first order, pseudo-second order, Elovich and intra-particle diffusion kinetic models. Because of the dependence on only one axis, the linear form was unsuitable for kinetic modelling.^75^ A significant number of literature reports have indicated that the non-linear forms of kinetic models are best suited to explain the adsorption study as compared to linear forms.^76,77^ Therefore, the non-linear form of the models was applied for all experiments.

The origin forms of Lagergren's pseudo-first order equation and Ho's pseudo-second order equation are generally expressed as (respectively):^78^*q*_*t*_ = *q*_e_(1 − e^−*k*_1_*t*^)
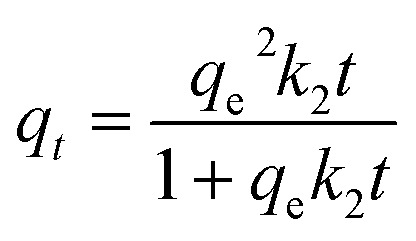
where *q*_*t*_ and *q*_e_ are the adsorption capacity (mg g^−1^) at any time *t* (min) and at equilibrium, respectively. The *k*_1_ and *k*_2_ are the pseudo-first order rate constant (min^−1^) and the pseudo-second order rate constant (g mg^−1^ min^−1^), respectively.

The important factors of the pseudo-second order model are initial rate constant (*h*) and half-life time (*t*_1/2_), which is the time needed to adsorb 50% of the amount of adsorbate that will be adsorbed at equilibrium. It can be calculated by the following equations:*h* = *k*_2_*q*_e_^2^
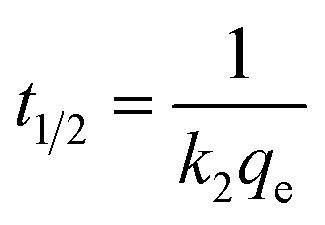


The Elovich equation is:
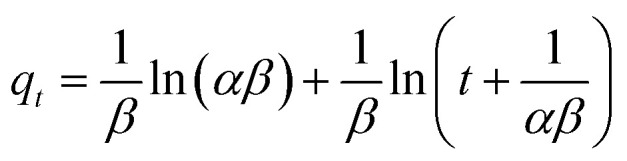
where *α* is the adsorption initial rate (mg g^−1^ min^−1^) and *β* is a constant (g mg^−1^) related to the external surface area and activation energy of adsorption (chemisorption).^79^

To compare the applicability of model equations and fitting them to the data, the correlation coefficient (*R*^2^), chi-square test (*χ*^2^) and the sum of the square of the errors (SSE) was calculated from:^78^
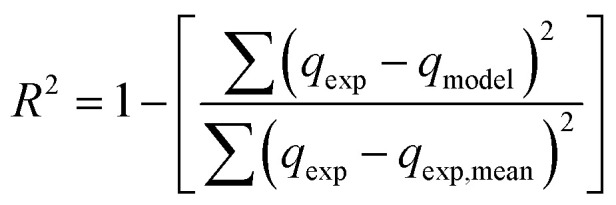

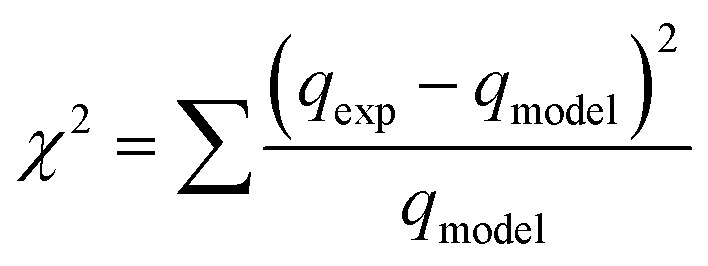
SSE = ∑(*q*_exp_ − *q*_model_)^2^where *q*_exp_, *q*_model_ and *q*_exp,mean_ are experimental values, model calculation and average experimental values of adsorption capacity (mg g^−1^), respectively.

For the best fit of kinetic model to be achieved in any adsorption study, three conditions should be satisfied: (1) the value of adsorption uptake from experiment (*q*_e,exp_) should reasonably match the value from model calculation, (2) the values of *R*^2^ should close to 1 and (3) the values of *χ*^2^ and SSE should be minimum.

From the values of *R*^2^, *χ*^2^ and SEE (Table 3), it was found that the pseudo-first order model match the experimental adsorption data of IBP on biochar, while pseudo-second order model demonstrated a better fit for magnetic biochar. Furthermore, the calculated adsorption values (*q*_e_) from the pseudo-first order model (125.79 mg g^−1^) and pseudo-second order model (115.57 mg g^−1^) were closely resemble the experimental adsorption capacity (*q*_e,exp_) for biochar and magnetic biochar, respectively. The constants *k*_1_ and *k*_2_ obtained from CB are higher than CMB indicating that the adsorption kinetics of biochar are quicker than that of magnetic biochar. Moreover, the initial adsorption rate (*h*) is 6.25 times faster in biochar compared to magnetic biochar. It was also found that the half-life period of CB is 0.303 min, while it is 1.730 min for CMB, thereby confirming the affinity of IBP to adsorb to the biochars and confirmed that biochar can adsorbed IBP faster than magnetic biochar. The Elovich model is consistent with the kinetic experimental data, *R*^2^ equal to 0.996 and 0.976 for CB and CMB, respectively. The Elovich parameters (*α*) showed high adsorption initial rate of IBP for both biochars. Importantly, the fast adsorption of IBP is one of the most attractive advantage of the new biochars produced in this study.

**Table 3:** Kinetic model parameters for the adsorption of IBP on biochars

Kinetic model	Parameter	Biochar	Magnetic biochar
—	*q*_e,exp_ (mg g^−1^)	124.05	114.26
Pseudo-first order	*q*_e_ (mg g^−1^)	125.79	111.31
	*k*_1_ (min^−1^)	0.555	0.255
	*R* ^2^	0.998	0.992
	*χ* ^2^	0.189	0.862
	SSE	23.719	88.196
Pseudo-second order	*q*_e_ (mg g^−1^)	126.74	115.57
	*k*_2_ (g mg^−1^ min^−1^)	0.026	0.005
	*h* (mg g^−1^ min^−1^)	417.639	66.782
	*t*_1/2_ (min)	0.303	1.730
	*R* ^2^	0.998	0.997
	*χ* ^2^	0.250	0.286
	SSE	31.321	30.835
Elovich	*α* (mg g^−1^ min^−1^)	6.005 × 10^42^	3.363 × 10^6^
	*β* (g mg^−1^)	0.814	0.155
	*R* ^2^	0.996	0.976
	*χ* ^2^	0.487	2.667
	SSE	60.757	274.965
Intra-particle diffusion	Stage I		
	*k*_id_ (mg g^−1^ min^−1/2^)	3.373	10.74
	*C* (mg g^−1^)	111.57	61.74
	*R* ^2^	0.929	0.962
	Stage II		
	*k*_id_ (mg g^−1^ min^−1/2^)	0.180	1.473
	*C* (mg g^−1^)	125.03	102.55
	*R* ^2^	0.938	0.981
	Stage III		
	*k*_id_ (mg g^−1^ min^−1/2^)	0.119	0.234
	*C* (mg g^−1^)	126.74	110.10
	*R* ^2^	0.935	0.925

**Fig. 12:**
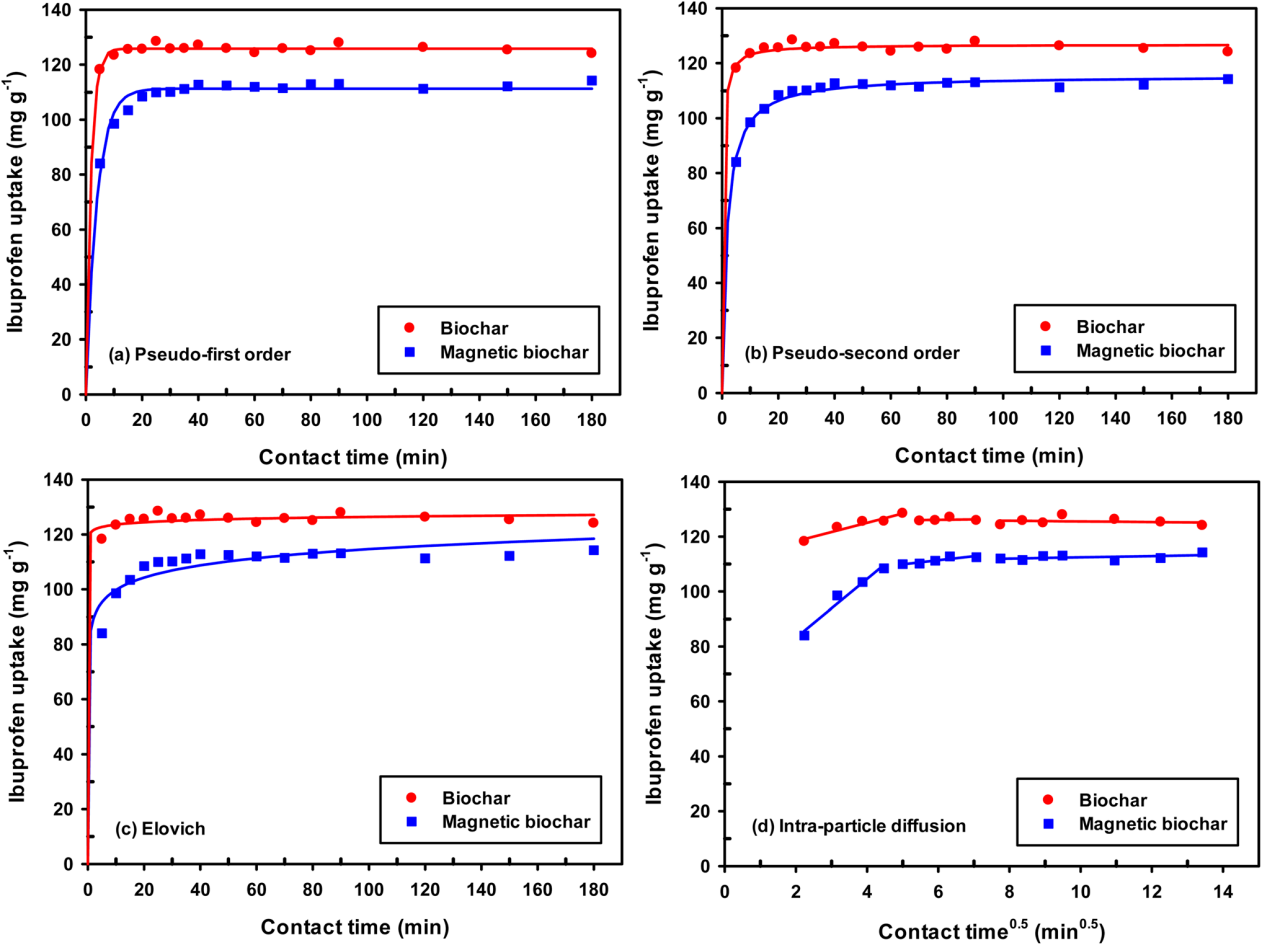
Kinetic adsorption curves (a) pseudo-first order, (b) pseudo-second order, (c) Elovich and (d) intra-particle diffusion model.

For better insight on the rate-limiting step, external transport (film diffusion) or intra-particle diffusion, the intra-particle diffusion model was fitted to the experimental results. This model is described as follows:*q*_*t*_ = *k*_id_*t*^0.5^ + *C*where *k*_id_ is the intra-particle diffusion rate constant (mg g^−1^ min^−1/2^) and *C* is intercept (mg g^−1^) which represents the thickness of boundary layer.^9^ According to Fig. 12(d), there are three separate stages: the first stage involving diffusion of the IBP molecules through the solution to the external surface of biochar or external diffusion, the second stage is the intra-particle diffusion effects IBP molecules through biochar pores and the third stage is the final equilibrium of adsorption. In addition, the plot for this model did not pass through the origin, indicating that the intra-particle process of IBU onto both biochars was not the only rate-limiting step, but the adsorption rate was also influenced by other mechanisms such as film diffusion and adsorption of the IBP molecules on the interior of the biochars pores. The values of the parameters and *R*^2^ obtained from this model for all three stages are also reported in Table 3. The values of *C* are all non-zero, confirmed that the plot did not pass through the origin. For both biochars, the values of *k*_id_ for stage I is greatest, which suggested that the external diffusion stage faster than intra-particle diffusion stage and equilibrium adsorption stage.

#### 3.2.2 Effect of pH on the adsorption

**Fig. 13:**
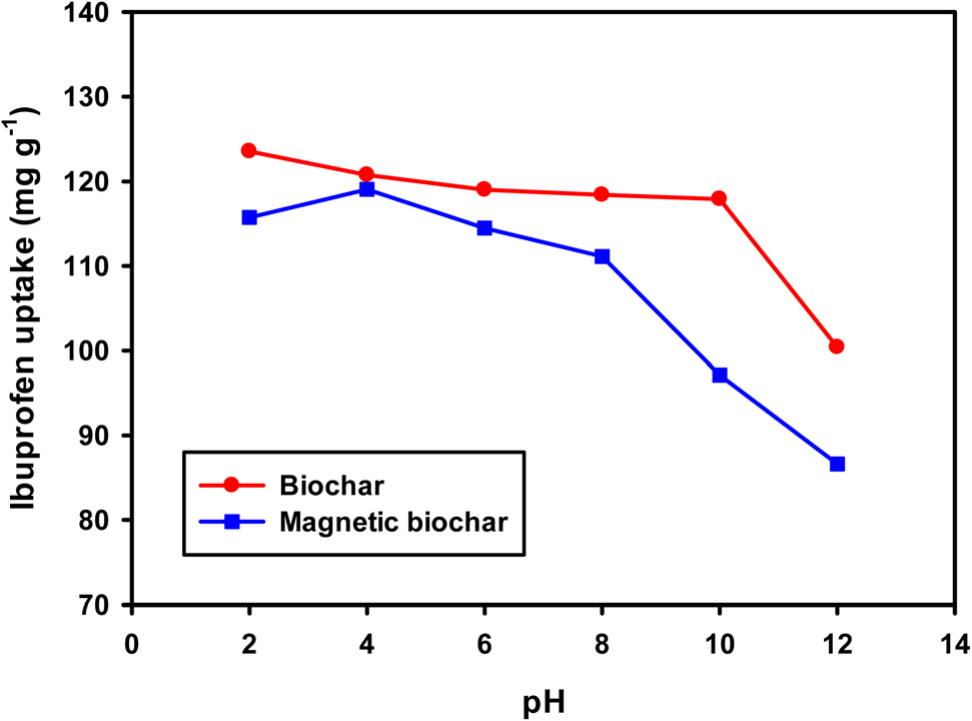
Effect of pH on the removal of IBP (room temperature; *C*_o_ = 30 mg L^−1^; time = 60 min; dosage 0.01 g/50 mL).

The effect of solution pH on IBP adsorption was investigated for both biochars (Fig. 13). For non-magnetic biochar, the adsorption uptake decreased when the pH was increased from 2 to 12. The adsorption uptake increased with the increase of solution pH from 2 to 4 for magnetic biochar, thereafter, the uptake decreased. Adsorbent surface charge (pH_pzc_) and p*K*_a_ of IBP with value of 4.9 were two factors affecting pH phenomena. As discuss before, the pH_pzc_ was 2.40 and 4.40 for biochar and magnetic biochar, respectively. A positive charge extended onto their surfaces when solution pH < pH_pzc_ and a negative charge developed when solution pH > pH_pzc_. In general, the IBP existed in water as two species: molecular (IBP) and anionic (IBP^−^). The rate of adsorption decreased when solution pH was more than p*K*_a_ value of IBP because more anionic form of IBP and more negative charge or less positive charge of biochars surface, leading to lower electrostatic interaction between adsorbate and adsorbents.^28^ When considering these results, the pH of 2 and 4 were adopted for adsorption isotherm study for biochar and magnetic biochar, respectively.

#### 3.2.3 Adsorption isotherms

For a better understanding of the adsorption mechanism, adsorption isotherms were constructed for both biochars and analysed using three non-linear isotherm models, namely Langmuir, Freundlich and Langmuir–Freundlich models. These models can be represented by:
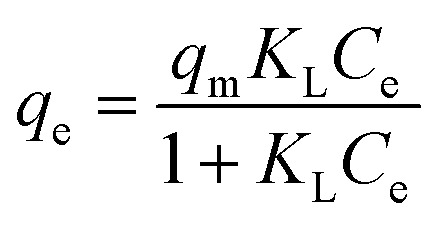
*q*_e_ = *K*_F_*C*_e_^1/*n*^
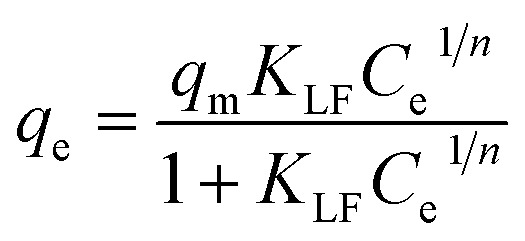
where *q*_e_ is the amount of IBP adsorbed at equilibrium (mg g^−1^); *C*_e_ is the equilibrium concentration of IBP in aqueous solution (mg L^−1^); *q*_m_ is the maximum adsorption capacity (mg g^−1^); *K*_L_ (L mg^−1^), *K*_F_ ((mg g^−1^)(L mg^−1^)^1/*n*^) and *K*_LF_ ((L mg^−1^)^1/*n*^) are the Langmuir, Freundlich and Langmuir–Freundlich constants, respectively; 1/*n* is the dimensionless parameter for Freundlich model related to adsorption intensity, which indicates the magnitude of the adsorption driving force or surface heterogeneity. Adsorption is favourable when 1/*n* < 1, unfavourable when 1/*n* > 1, linear when 1/*n* = 1 and irreversible when 1/*n* = 0.^80^

Moreover, the separation factor (*R*_L_) which determines the nature of the isotherm shape is an important feature of the Langmuir model. This dimensionless parameter is given by:
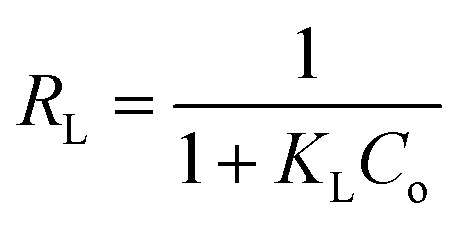
where *C*_o_ is the initial IBP concentration (mg L^−1^). Adsorption is favourable when 0 < *R*_L_ < 1, unfavourable when *R*_L_ > 1, linear when *R*_L_ = 1 and irreversible when *R*_L_ = 0.

**Fig. 14:**
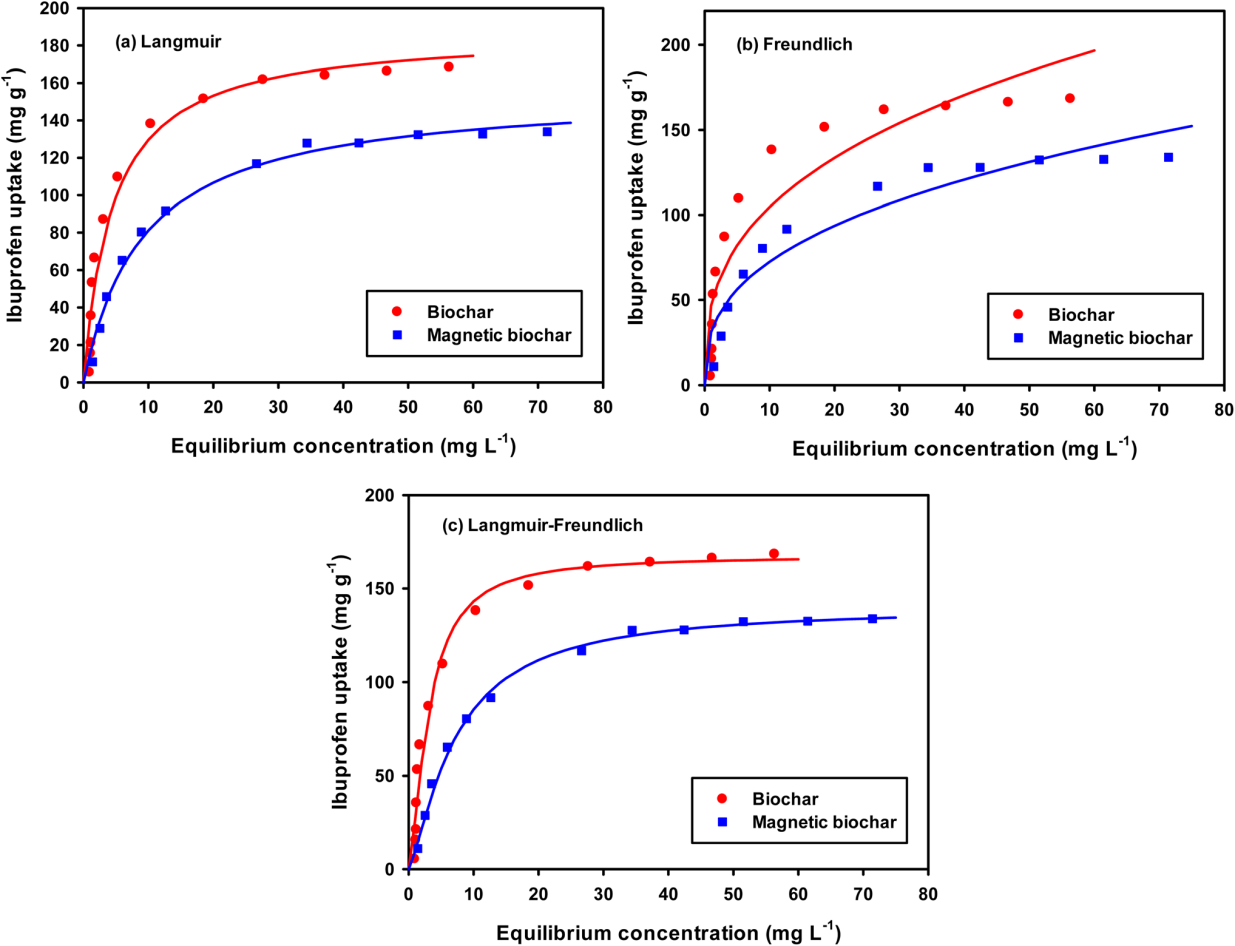
Equilibrium isotherms of IBP adsorption (room temperature; *C*_o_ = 5–100 mg L^−1^; time = 60 min; pH = 2 for CB and pH = 4 for CMB; dosage 0.01 g/50 mL). The symbol represents experimental data and solid line represents fitting of (a) Langmuir, (b) Freundlich and (c) Langmuir–Freundlich models.

Fitting of the experimental data to the isotherm models are shown in Fig. 14 and parameters as well as error functions are presented in Table 4. The highest value of *R*^2^ and lowest values of *χ*^2^ and SSE for both biochars was through the application of the Langmuir–Freundlich model on the experimental data. This indicated that two distinct adsorption mechanisms can occur: homogeneously with monolayer formation following the Langmuir model and also heterogeneously with multilayer adsorption corresponding to Freundlich model. The maximum adsorption capacities (*q*_m_) were 167.51 and 140.50 mg g^−1^ for non-magnetic and magnetic biochars, respectively. The calculated *R*_L_ values for adsorption of IBP are also presented in Table 4. It is observed that *R*_L_ values for both biochars are between 0 and 1, indicated favourable adsorption. These findings are in good agreement with the 1/*n* value obtained from Freundlich model (<1), which also suggested that adsorption was favourable.

**Table 4:** Adsorption isotherm parameters for the adsorption of IBP on biochars

Model	Parameter	Biochar	Magnetic biochar
Langmuir	*q*_m_ (mg g^−1^)	187.46	155.70
	*K*_L_ (L mg^−1^)	0.224	0.109
	*R*_L_	0.043–0.309	0.084–0.648
	*R* ^2^	0.956	0.988
	*χ* ^2^	51.775	6.259
	SSE	2076.217	206.378
Freundlich	*K*_F_ ((mg g^−1^)(L mg^−1^)^1/*n*^)	46.509	31.072
	1/*n*	0.352	0.368
	*R* ^2^	0.844	0.904
	*χ* ^2^	109.784	31.856
	SSE	7129.363	1823.383
Langmuir–Freundlich	*q*_m_ (mg g^−1^)	167.51	140.50
	*K*_LF_ (L mg^−1^)^1/*n*^	0.184	0.072
	1/*n*	1.504	1.331
	*R* ^2^	0.975	0.997
	*χ* ^2^	37.442	2.350
	SSE	1295.884	98.631

To validity the adsorbent properties of both biochars derived from chrysanthemum waste, their adsorption capacities must be compared with other adsorbents reported in literature. Table 5 shows the values of maximum adsorption capacity, surface area of adsorbent, time to reach equilibrium and optimum pH for the adsorption of the present study compared with other biochar materials reported in the literature. It is evident that adsorption capacity of chrysanthemum biochar and magnetic biochar which is higher than a significant number of other biochar materials reported in the literature. Moreover, the adsorption process for the biochars from this current study are both rapid and simple compared to the methods employed for many other adsorbents. The optimum pH values for adsorption agree with previously published research. However, the effect of adsorption temperature should be performed in future work to study free energy, enthalpy change and entropy change. Moreover, the competitive adsorption between IBP and ions in actual water bodies such as Ca, Mg or Na should also be studied in future.

**Table 5:** Comparison of maximum adsorption capacity for IBP on various type of adsorbents

Adsorbent	Surface area (m^2^ g^−1^)	Time for equilibrium (min)	pH of solution	Adsorption capacity (mg g^−1^)	Reference
Chrysanthemum biochar (CB)	220	60	2	167	This current study
Chrysanthemum magnetic biochar (CMB)	194	60	4	140	This current study
Magnetite/multiwall carbon nanotube functionalized with hydrazine	187	15	4	12	15
Steam activated sugarcane bagasse biochar	—	360	2	12	28
H_3_PO_4_ activated sugarcane bagasse biochar	—	360	2	14	28
Steam activated mung bean husk biochar	—	120	2	60	26
Activated walnut shell biochar	686	1440	4	70	24
Chemically modified N-biochar from *Parthenium hysterophorus*	—	120	4	90	81
Carbon nitride (C_3_N_4_)/soot composite	60	1440	—	149	82
Rice straw biochar	76	40	—	170	22
*Alternanthera philoxeroides*-based biochar	858	—	—	172	23

## 4 Conclusions

For the first time, biochar production using chrysanthemum waste from the beverage industrial was investigated and applied to the removal of IBP from aqueous solutions. Magnetic biochar was also produced with ferric chloride (FeCl_3_). FTIR, TGA, XPS, XRD, EDX and VSM analysis confirmed incorporation of iron leading to a magnetic biochar. The obtained biochar and magnetic biochar had BET surface area of 220 and 194 m^2^ g^−1^, respectively. The adsorption process parameters were investigated using the batch mode experiments. The results from kinetic studies executed non-linear pseudo-second order kinetic model as the best-fitted model for both biochars. Adsorption results demonstrated that the IBP removal by both biochars are highly dependent on the pH of the solution, with maximum adsorption capacities at pH 2. Langmuir–Freundlich isotherm model fitted well with the experimental data of the adsorption process, with maximum uptake capacities of 167 and 140 mg g^−1^ for biochar and magnetic biochars, respectively. Although magnetic biochar had a lower surface area and adsorption capacity of IBP than the non-magnetic biochar, however an advantage is that it can be easily separated from solution. Results revealed that the chrysanthemum biochars are efficient adsorbent and could possibly be used for the remediation of pharmaceutical drugs from wastewater. The reduction of voluminous bio-based industrial waste to produce a value-added biochar through pyrolysis provides possible ways to management and efficient utilise this waste.

## Author contributions

Y. N. designed the conception, wrote the manuscript, made calculation, plots all figures, and discussed the results; D. P., K. S., K. K., P. P. and R. P. performed the experiments; A. J. H. performed SEM-EDX experiments, corrected English language and discussed the results.

## Conflicts of interest

There are no conflicts to declare.

## Supplementary Material
